# Using Manual and Computer-Based Text-Mining to Uncover Research Trends for *Apis mellifera*

**DOI:** 10.3390/vetsci7020061

**Published:** 2020-05-06

**Authors:** Esmaeil Amiri, Prashant Waiker, Olav Rueppell, Prashanti Manda

**Affiliations:** 1 Department of Biology, University of North Carolina at Greensboro, Greensboro, NC 27402, USA; p_waiker@uncg.edu (P.W.); o_ruppel@uncg.edu (O.R.); 2 Department of Computer Science, University of North Carolina at Greensboro, Greensboro, NC 27402, USA; p_manda@uncg.edu

**Keywords:** text-mining, topic modeling, colony collapse disorder, genomics, *Varroa* mite, honey bee health, *Apis mellifera*

## Abstract

Honey bee research is believed to be influenced dramatically by colony collapse disorder (CCD) and the sequenced genome release in 2006, but this assertion has never been tested. By employing text-mining approaches, research trends were tested by analyzing over 14,000 publications during the period of 1957 to 2017. Quantitatively, the data revealed an exponential growth until 2010 when the number of articles published per year ceased following the trend. Analysis of author-assigned keywords revealed that changes in keywords occurred roughly every decade with the most fundamental change in 1991–1992, instead of 2006. This change might be due to several factors including the research intensification on the *Varroa* mite. The genome release and CCD had quantitively only minor effects, mainly on honey bee health-related topics post-2006. Further analysis revealed that computational topic modeling can provide potentially hidden information and connections between some topics that might be ignored in author-assigned keywords.

## 1. Introduction

Western honey bees, *Apis mellifera*, are of interest due to their beneficial products and impact on food security [1,2]. Their role as general pollinators and the easy mobility of large colonies with thousands of worker bees make them an indispensable component of modern agricultural systems [2,3]. Furthermore, they are an attractive scientific model to study caste development, haplo-diploidy, eusociality, symbolic language, and many other fundamental scientific topics [4,5].

Although the number of honey bee colonies has increased on a global scale, the demand for the pollination service of cultivated crops along with high overwintering colony mortality and changes in the political and socioeconomic system threaten the sustainability of local honey bee industries [6,7,8,9,10]. The decline in the population of pollinators has been of concern for stakeholders since the 1990s. In 2006, the description of a novel honey bee health problem called colony collapse disorder (CCD) allegedly caused a surge in research to understand and overcome this calamity [11,12]. Concomitantly, the release of the sequenced genome of *Apis mellifera* [13] facilitated new tools, which may have triggered a surge in fundamental research on honey bee health and biology [14]. However, the quantitative and qualitative consequences for the scientific output of honey bee research and sub-disciplines are unclear. A comprehensive aggregation of scientific knowledge to explore deep insights into apicultural research trends is lacking, which have been shown for other sub-fields of science [15,16]. It was estimated that the overall annual growth of scientific peer-reviewed articles was at a rate of 8–9 percent in recent years [15]. This volume of scientific output can exceed the capacity of researchers to keep track of every single publication and keep up with the pace of change in research within their field of expertise. Although review articles can effectively sum up the current state of research on a particular topic, they have been frequently criticized for presenting only a narrow and subjective view instead of a comprehensive update on the research field [17,18,19]. Moreover, connections and relationships between research topics are often overlooked by the reader because papers can contain valuable hidden knowledge beyond their key findings [20,21]. The development and application of text-mining tools and machine learning algorithms have been on the rise to address these limitations [15,16,19,20]. Text-mining tries to reduce the human role and instead utilize functional automated or semi-automated tools allowing researchers to evaluate the large volume of existing literature in an efficient manner [16,22,23].

In this study, we estimated overall research publication growth on *Apis mellifera* and tested the hypothesis that the release of the honey bee genome and the report of colony collapse disorder had a significant impact on shifting the focus of honey bee research. First, we extracted author-assigned keywords associated with the honey bee literature and analyzed them to understand usage and connections between different keywords over time. Next, we analyzed trends in honey bee research before and after 2006 to test our hypothesis. We also explored temporal trends for certain keywords of interest and investigated differences in keyword prevalence before and after 2006. We complemented these manual efforts with automated topic-modeling that used the abstract and title as additional data sources to compare manual and computer-based analyses of the literature.

## 2. Methods

### 2.1. Dataset

The Scopus database (www.scopus.com) was queried on 9 June 2018 using the search term “apis mellifera”. All publications with the search term in the title, abstract, or keywords were retrieved as a subset of the honey bee literature. A minimum threshold of five publications per year led to the inclusion of all years from 1957 to 2017. Due to incomplete coverage and indexing at the time of retrieval, the data from 2018 was not included.

We further selected two different approaches to analyze this dataset. For author-assigned keyword analysis, we only used keywords, while for computer-based keyword analysis, we used the combination of keywords, abstracts, and titles.

### 2.2. Author-Assigned Keywords’ Analysis

#### 2.2.1. Pre-Processing the Dataset

Most of the retrieved publications from Scopus contained a set of author-assigned keywords. These keywords were pre-processed via stemming and manual synonymization to remove redundancies due to lexical differences across different publications. Initial manual exploration of keywords across publications revealed that authors often used synonyms of the same word. We manually assigned the keywords to a consistent synonym where applicable. For example, “honey-bee (apis mellifera)”, “honey-bee apis mellifera”, “honey bee a. mellifera”, and “honey bee apis mellifera” were found as keywords in different publications. These keywords were all assigned “apis mellifera” as the synonym. We limited the manual synonymization process to the set of keywords that occurred more than once in the full dataset (publications from 1957–2017). Further, syntactic issues among the keywords such as different forms of the same keyword (e.g., singular and plural) were addressed by stemming keywords using the Porter Stemmer [24]. Stemming is a process that reduces a word to its etymological root, thereby removing redundancies due to different forms of the same root. For example, the words “foraging” and “forage” stem to” forag”. Similarly, the words “colony” and “colonies” stem to “coloni”. The final set of synonymized keywords were analyzed using multiple techniques, as described in subsequent sections.

#### 2.2.2. Temporal Trends

The temporal trends of all keywords were examined to identify the change of interest in specific research areas over time. For each keyword, we analyzed the proportion of publications containing the particular keyword in each year of the dataset.

#### 2.2.3. Cluster Analysis

Individual years in the data were clustered based on their similarity in keyword frequency to test whether 2006 represented a fundamental change in honey bee publication patterns. To analyze differences before and after 2006, the dataset of synonymized keywords was separated into two datasets corresponding to the time periods of (1) 1957–2005 and (2) 2006–2017. The top 50 most frequently occurring keywords were selected from each of the two time periods. After removal of the search term “apis mellifera”, the remaining 49 keywords from each period were combined, resulting in 65 unique keywords that were used for the subsequent cluster analysis. Cluster analysis was performed by hierarchical clustering (R *hclust* function) with the Ward distance method (R “*ward.D*” method) [25]. To validate the clustering, we performed bootstrapping on our data for 1000 iterations using the R package “fpc” [26]. The keyword distribution over the resulting clusters was analyzed by creating a heatmap. The “RColorBrewer” package was further applied for better visualization and color enhancement. All the operations were performed in the RStudio v1.0.143 environment [27]. To identify dominant research topics across each cluster, we calculated the expected frequency for each keyword, based on the marginal sums (across all keywords for each year and for each keyword across all years) and then built a ratio (observed/expected). For each cluster, the geometric mean of these ratios was calculated (transformed by adding 0.001 to avoid division by zero) for each keyword across all years in the cluster. The three keywords with the highest observed/expected ratio were selected to illustrate dominant research topics of each cluster.

#### 2.2.4. Network Analysis

Networks illustrating the representation and co-occurrence of keywords were built and analyzed to explore connectivity among the common keywords. Two networks were created using the Gephi visualization software v0.9.2 [28] based on the top 49 keywords from the two time periods; 1957–2005 and 2006–2017. Each node represents a unique keyword and connections (edges) between two keywords were drawn if they co-occurred together in more publications than expected by chance according to the following calculation:

Consider keywords *i* and *j*. Keyword pairs where the observed co-occurrence probability (*O*) is greater than the expected co-occurrence probability (*E*) are connected via an edge. *O* is defined as the number of publications containing both *i* and *j* as keywords. *E* is defined as *p*(*i*) * *p*( *j*) where *p*(*i*) is the probability of observing keywords *i* and *j*, respectively. Edge weights are proportional to *E – O*; the thickness of the connections represents the difference between the expected and observed probabilities of co-occurrence: a higher thickness depicts a higher observed co-occurrence.

The transitivity (known as the clustering coefficient) was computed on keyword networks generated from both time periods to explore differences in edge density. Transitivity is a measure of the degree to which nodes in a graph tend to cluster together [29]. It was computed using the density of triplets of nodes in a network graph [30]. Transitivity was calculated using the *transitivity()* function of the R package “igraph” [31]. To validate the results of transitivity, 95% CI were calculated by bootstrapping over 1000 iterations for each dataset using a custom R script.

### 2.3. Computer-Based Keywords’ Analysis

#### 2.3.1. Preparing and Pre-Processing the Corpora

According to the previously described hypothesis, two different corpora were built to conduct topic modeling from articles retrieved via Scopus: (1) pre-2006, which contained data (title, keywords, and abstract) for 5640 articles published between 1957 and 2005, and (2) post-2006, which contained data (title, keywords, and abstract) for 8473 articles published between 2006 and 2017. Each corpus was pre-processed using the steps of (1) corpus cleaning in which scientific notations, special characters, punctuation, and numbers were removed and (2) stemming, performed using the Porter Stemmer [24].

#### 2.3.2. Applying Topic Modeling

Topic modeling is a text-mining technique to analyze large volumes of text to discover latent topics and patterns within texts. We applied latent Dirichlet allocation (LDA) [21] to both of the corpora. LDA assumes that the topic distribution has a sparse Dirichlet prior that supports the intuition that documents consist of a mixture of topics and that these topics can be described using sets of relevant words. One of the inputs to the LDA topic model is the number of topics to be generated from the corpus. Selecting the optimal number of topics from any corpus is a problem that has received extensive attention. As a result, several automated metrics such as perplexity [32] and coherence [33] have been developed to evaluate topic models. Several studies report that inferences of topic models’ quality based on perplexity were negatively correlated with human perception [34,35]. Recent work [33] suggested coherence to be a measure that aligns better with the human perception of a model’s quality.

We used a two-pronged approach to select the appropriate number of topics to be generated from the corpora. We developed models with 20, 50, 70, 90, 110, and 140 topics and evaluated the coherence scores using the *c_v_* coherence metric [36]. Subsequently, the topics were manually inspected by domain scientists on our team to select the most representative model for the data. Based on these metrics, the model with 20 topics was selected for further analyses in this study. The topic models were analyzed/visualized as follows:

Overall view using LDAvis: First, the topic models were presented using LDAvis [37], a web-based interactive visualization tool. The visualization provided an overview of all topics while highlighting the important words associated with each topic. To confirm that this automated protocol yielded a meaningful grouping of the words, we evaluated the top ten words in each topic and manually labeled them to the most appropriate research focus they represented. LDAvis allows the user to glance over individual topics while keeping the entire topic landscape in view and is thus helpful to the user when interpreting and labeling topics.

Network analysis of topics: Next, we created two networks, using the visualization software, Gephi, populated by the top 5 words associated with each of the 20 topics in both time periods. These networks indicated the important words and scientific areas in each period as inferred by the topic modeling algorithm. Nodes in each network are important words for a topic, and edges connect words that co-occur in a topic. Further, to compare the density of interconnection in the network plots, transitivity analysis was performed as described in the previous section.

Comparison between the topic model and keyword networks: To make a comparison of human (keywords) versus automated methods (topic modeling), networks built using the two approaches for each time period were compared to estimate overlaps and differences. The networks were compared to identify overlaps in nodes and edges. An overlap in nodes was noted if the same node was present in both networks or if a node in one network was a substring in the other. Similarly, an overlapping edge was identified in one of three cases: (1) an edge between the same pair of nodes was present in both networks; (2) an edge between a node pair with one node matched exactly, and the other was a substring; and (3) an edge between a node pair where both nodes were related via substrings.

## 3. Results

### 3.1. Dataset

**Figure 1:**
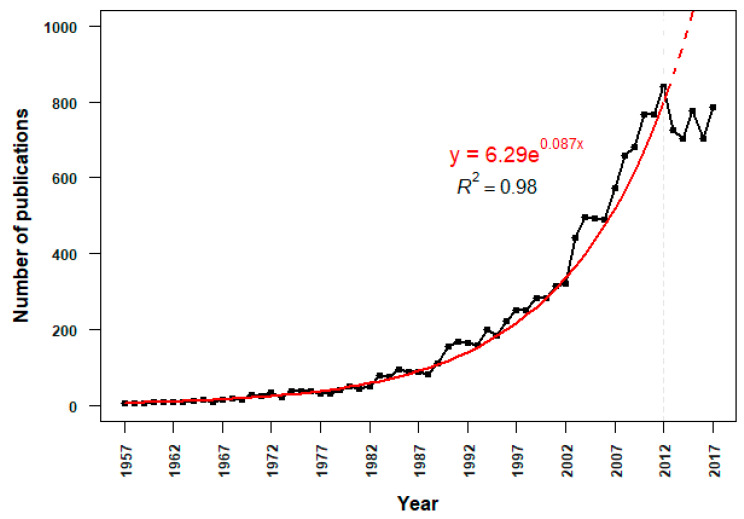
Publication trends over time for research articles retrieved from Scopus using the search Table 1957 to 2017. The publication growth fit an exponential regression curve until 2010, after which the growth leveled off with the exception of 2012.

The Scopus search resulted in 14,113 articles with publication years ranging from 1957 to 2017. The publication growth could be approximated (R^2^ = 0.98, n = 55, *p* < 0.001) by the exponential function ($y=6.29\;e^{0.087x}$), with the number of publications doubling every 7.97 years. The exponential growth continued until 2010, after which the number of retrieved publications did not further increase consistently (Figure 1).

### 3.2. Author-Assigned Keywords’ Analysis

#### 3.2.1. Temporal Trends

In general, the temporal trends of relative keywords abundance followed one of three trends: (1) general exponential increase, similar to the total number of publications; (2) abrupt increase without any prior occurrences; (3) increase with subsequent decrease. The average occurrence of most keywords increased with time, although the smaller number of publications per year in earlier years caused some large fluctuations. We selected a few keywords associated with two research foci “health” and “genomics” to highlight these temporal trends (Figure S1).

#### 3.2.2. Cluster Analysis

**Figure 2:**
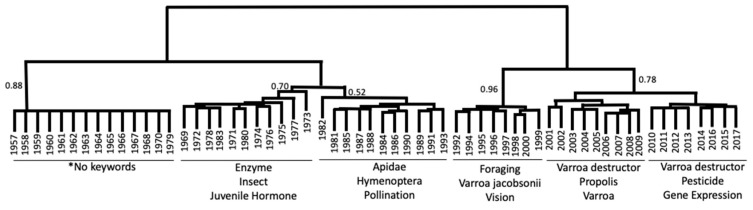
Cluster analysis of years based on the relative frequency of the overall most common aggregated keywords. Years clustered approximately into separate decades, but the most fundamental division in the dataset occurred between 1991 (and prior years) and 1992 (and following years). Bootstrap support is given for the major clusters. Furthermore, we show the three most enriched keywords for each cluster. * The most common keywords were not used in early articles, and therefore, the leftmost cluster did not have any over-enriched terms among the top keywords.

Publication years were clustered based on similar usage of the 65 most common keywords prior to and after 2006 to evaluate the overall change in research focus over time (Figure 2). As shown in Figure 2, the most fundamental split among years occurred between 1991 and 1992 with the exception of 1993. The sub-clusters in both time periods corresponded roughly to decades. Moreover, the usage of words was plotted over the year cluster using a heatmap to explain the basis of clustering (Figure S2). Most top keywords were absent in the early years. The top keywords started to show up during the 1970s, and the years from 1992 to 2017 showed the highest frequency of these words (Figure S2).

#### 3.2.3. Network Analysis

**Figure 3:**
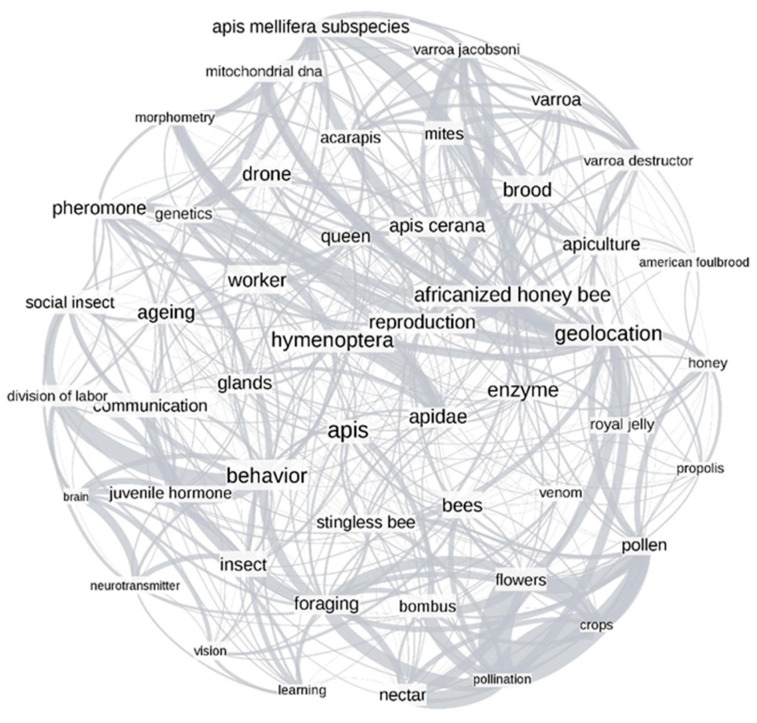
Network analysis plot of the top 49 keywords over the time period 1957–2005. The font size shows the degree of connections to that particular keyword, while each connection between words denotes a significant co-occurrence of the words in a research article. The edge thickness shows how much more keywords co-occur than expected by chance.

**Figure 4:**
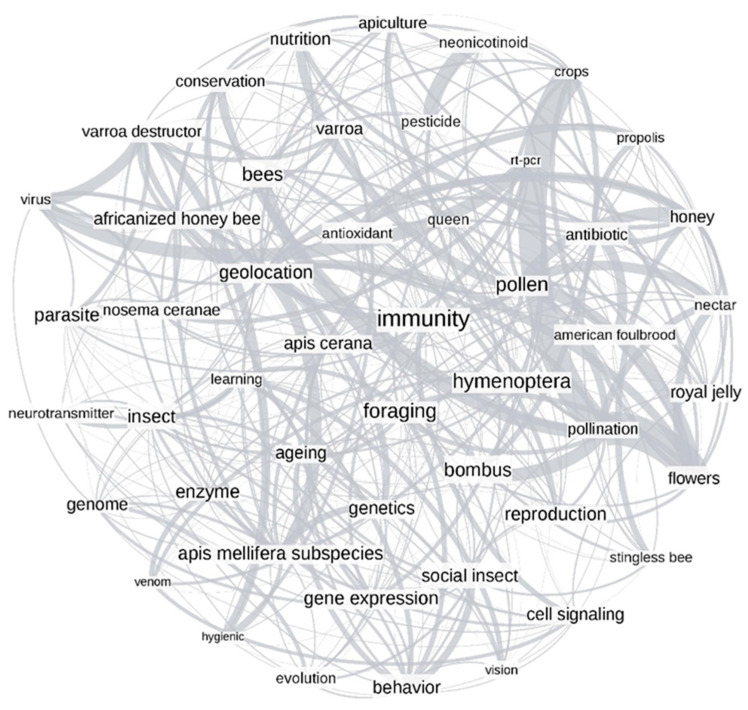
Network analysis plot of the top 49 keywords over the time period 2006–2017. The font size shows the degree of connections to that particular keyword, while each connection between words denotes a significant co-occurrence of the words in a research article. The edge thickness shows how much more keywords co-occur than expected by chance.

Keyword networks were generated for the time periods of 1957–2005 (Figure 3) and 2006–2017 (Figure 4). Changes in the size of several words, signifying the degree (number of connections within the network) implied that the connections between topics in honey bee science were dynamic. Time changes were further confirmed by the presence of unique top keywords in each time period. For example, the words “behavior”, “enzyme”, and “brood” in Figure 3 had more connections to other words in the plot, while words such as “vision”, “brain”, and “neurotransmitter” had lesser connections. We noticed substantial changes in the same words in the post-2006 time period (Figure 4). For instance, words such as “immunity”, “pollen”, and “foraging” had more connections, while the words “venom”, “hygienic”, and “vision” had fewer connections. Most of the words were interconnected in both plots, which implied the significant co-occurrence of any set of two words more often than expected by chance. The measure of global clustering in the network (called transitivity) was higher in the period of 1957–2005 (transitivity, T = 0.53) suggesting higher connection density than in the later period (T = 0.44). The confidence intervals for these transitivity values were determined by bootstrapping over 1000 iterations (Table S1), and no overlap between confidence intervals of these transitivity values suggested that they were significantly different from each other. While the relative co-occurrence of some pairs did not change between time periods (e.g., “pollination - flowers” and “pollination - crops”), most highly over-represented connections changed (e.g., “behavior - division of labor” in Figure 3 and “pesticide - neonicotinoid” in Figure 4).

### 3.3. Computer-Based Topic Modeling

The 20 topics generated from computer-based topic modeling for the two time periods can be explored by the reader in detail using the interactive visualizations available within our data deposit (see the Supplementary Materials). A snapshot of the visualization for the pre-2006 period is shown in Figure S3.

Manual analysis of the 20 topics indicated the presence of research themes such as “pollination”, “genomics”, “behavior”, “reproduction”, “apiculture”, “varroa infestation”, etc. The largest topic pre-2006 contained words such as “bee, forage, dance, test, model, pattern, fruit, communication, language, discrimination”, indicative of research in behavior. In contrast, the largest topic post-2006 contained words such as “bee, geolocation, pollen, honey bee, apiculture, colony, hive, collect, year, nutrition”, indicative of research in apiculture. The relevant words for each topic also were manually analyzed to label topics with research sub-fields where possible. For example, Topic 6 in the pre-2006 time period contained words such as “queen, pheromone, egg, reproduction, larvae, gland, ovary, cast, produce, cell” (Table S2). These words were labeled as “reproduction”. Similarly, we identified topics corresponding to “behavior”, “pollination”, “varroa”, and “genomics” pre-2006 and topics corresponding to “nosema infection”, “population”, and “virus infection” post-2006. Some sub-fields such as “genomics” were observed both pre- and post-2006. While certain topics were coherent and clearly indicative of a specific research area, other topics were deemed by the human observer (the authors) as a mixture of words from different areas and could not be labeled with a specific research theme. Tables S2 and S3 show five example topics corresponding to specific research foci from the two time periods.

**Figure 5:**
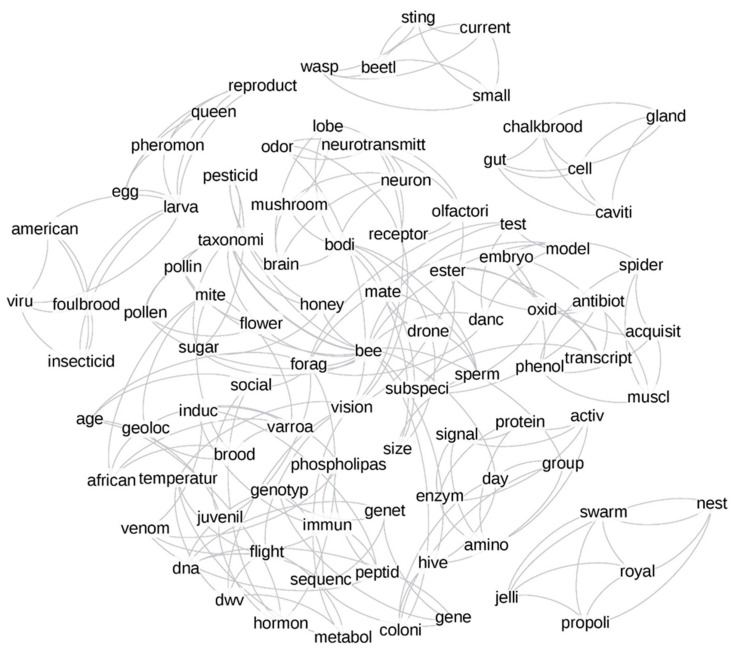
Topic modeling network graph of the top five words from each topic for the period of 1957–2005. The connection between words represent the co-occurrence of the words together in the title, keywords, and abstract of the analyzed articles.

**Figure 6:**
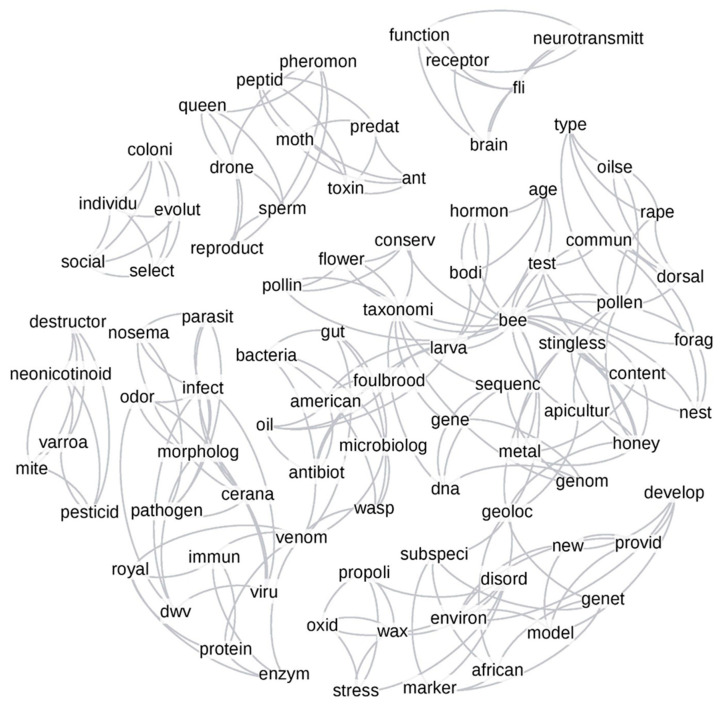
Topic modeling network graph of the top five words from each topic for the period of 2006–2017. The connection between words represents the co-occurrence of the words together in the title, keywords, and abstract of the analyzed articles.

The top five relevant words from each topic were used to create networks to shed light on the overall patterns of the co-occurrence and interconnection of computer-generated keywords before and after 2006 (Figure 5 and Figure 6). The top five words from each topic were automatically connected to each other and formed a small sub-cluster. These small sub-clusters could be connected to each other through common words among themselves, forming larger clusters in the graph. For example, in Figure 5, words such as “bee”, “forage”, “dance”, “test”, and “model” from Topic 1 formed a small sub-cluster, which was connected to the small sub-cluster formed by the words “bee”, “coloni”, “hive”, “day”, and “group” from Topic 2 through the common word “bee” and even making a larger cluster by connecting to Topics 3, 4, and 16 by the same common word. Visual inspection revealed that important research connections in specific sub-fields were formed post-2006 as the field matured and new knowledge was gained. For example, the term “queen” was connected to only a few reproductive-related terms such as words “queen”, “reproductive”, “pheromon”, “larva”, and “egg” in the pre-2006 network (Figure 5), while neglecting the role of some important terms such as “drone” and “sperm”, which appeared in the later time period (Figure 6). Further, we observed more small independent clusters not connected to the rest of the network post-2006 (Figure 6): for example, a cluster with words “brain”, “neurotransmitt”, “fli”, “function”, and “receptor” and another cluster with “mite”, “varroa”, “destructor”, “neonicotinoid”, and “pesticid” were not connected to the rest of the network, indicating more specialized research areas in the later time period.

Similar to the author-provided keywords, the computer-generated network graphs exhibited a difference in connection density among sub-clusters: it was higher in the 1957–2005 period (transitivity, T = 0.80) than 2006–2017 (T = 0.71). The confidence intervals for these transitivity values were determined by bootstrapping over 1000 iterations (Table S1), and no overlap between confidence intervals of these transitivity values suggested they were significantly different from each other.

### 3.4. Author-Assigned vs. Computer-Based Networks

We compared networks from the author-assigned keyword (Figure 3 and Figure 4) to the topic modeling networks (Figure 5 and Figure 6) to explore similarities and differences between the human (keywords) and automated (topic models) methods. The overlap of network nodes between the topic network and keyword network was 44% and 51%, respectively, for pre- and post-2006, while only 23% and 21% of the edges in the topic model network overlapped with the keyword network pre- and post-2006, respectively. The higher topic overlap with keywords and only a fraction of edge overlap suggested that computer and human methods were more congruent in finding single topics than connections.

## 4. Discussion

Continuous discoveries and publications have inundated scientific repositories with a tremendous volume of research articles [38]. The volume of articles threatens to exceed human capacity to read, understand, and comprehend the findings.

Here, we applied text-mining to aggregate the current knowledge and investigate potential research trends in the research field literature related to *Apis mellifera*, using over fourteen thousand scientific articles published between 1957 and 2017 from the Scopus database. Scopus is described as a source-neutral database that curates data from 24,600 journals/conference proceedings spanning across 5000 publishers. Scopus was selected as the source of data collection since we found it to be the most comprehensive resource available in addition to the ease of use for computational applications [39], compared to PubMed and Microsoft Academic Database. We are aware that using a single data source might introduce bias in the retrieved articles, which depend on where researchers choose to publish or a change in the portfolio of journals the database has selected to archive. We retrieved the articles by limiting our search to the term “apis mellifera” and avoided the generic word “honey bee” because the term “honey bee” has been used redundantly for several species, and our intent was to analyze specifically literature on Western honey bees, which are present all over the world [8]. It should be noted that the publications chosen here for our analysis were restricted to those published in English or available in multiple languages, one being English. Scientific literature, particularly before 1957 and shortly after, was predominantly published in German and French. English slowly became the language of scientific communication. We acknowledge that this choice omits a subsection of literature published in other languages portraying a geo-linguistic focus of Western literature culture.

We found that the number of research publications in honey bee science is growing similar to other biological sub-fields [19]. This exponential growth pattern of publications was consistent until the year 2010 with an exceptional of the year 2012. There could be multiple causes for such a growth rate. It could be the increase in the number of scientists during this period [40], technological advancement in the research field [41], or the administrative pressure to get or remain in research positions at academic institutions [42]. Furthermore, part of it can be explained by the onset of publications related to the honey bee genome sequencing project, which was conceptualized during 1998–2001 in several courses, workshops, and conferences [43]. In addition, honey bees have been experiencing higher colony losses in the U.S. and Western Europe, but relatively fewer in other parts of the world since the year 2006, which led to a surge in honey bee health research in the following years [10,44,45]. The saturation toward later years could be the result of the field maturation after publishing mostly descriptive research, leaving larger scale projects and investigations that require greater effort, time, and resources. Alternatively, the leveling off of the publication rate after 2010 might be an artifact of using Scopus as the sole data source. The result may simply reflect shifts in author choices of publication venues or changes in Scopus’ publication sources.

We conducted cluster and network analyses on author-assigned keywords to understand trends, connectivity, and shifts in research sub-fields (Figure S2). In the scientific community and the public domain, it is commonly assumed that the discovery of CCD and release of the sequenced genome had a major impact on honey bee science, but this assumption was never tested. Our cluster analysis revealed that the usage of keywords experienced the biggest change in 1991–1992, and not in 2006. This observation might be due to several reasons including technological advancements [41], changes in political and socioeconomic systems [10], and the increasing impact of the parasitic *Varroa* mite [46,47]. There is no doubt that scientific advances depend not only on new ideas, conceptual leaps, and paradigm shifts, but also to a large extent on technology advances that make these steps possible. Technological advancements such as the emergence of PCR, genomic technologies [41], and the invention and use of the World Wide Web across academia are a notable few. These factors might have helped in elevating the research outputs and establishment of improved communications among researchers. Although the *Varroa* mite has been known and widespread in Europe since the 1970s [48], the final globalization of this honey bee health problem might be an explanation for our results [8,49]. It has been shown that research on the *Varroa* mite was by far the most studied bee threat in the early 1990s [50]. This was confirmed in our identification of major research topics across clusters that *Varroa* was a prominent research topic in all clusters from 1992 through 2017 (Figure 2). Our results also suggested that the main concepts and concerns of honey bee research had roughly changed every ten years (Figure 2). This might be due to the requirement and applicability of research; a shift in concepts and the development of advanced methodological tools, which often takes time to replace the earlier issues, and the duration may take up to a decade.

The co-occurrence of author-assigned keywords was explored using network analysis (Figure 3 and Figure 4). The abundance of connections in both networks (pre- and post-2006), showing greater than randomly expected co-occurrence, indicated that many keywords were grouped and did not occur randomly. In part, this might be explained by the paucity of keyword usage in the early years of our dataset. However, tight clusters of correlated keywords, such as the clusters related to pollination (“crops”, “flowers”, “nectar”, and “pollination”), colony products (“pollen”, “honey”, “royal jelly”, “venom”, and “propolis”), and taxonomical classifications (“insect”, “hymenoptera”, “bees”, “apis mellifera subspecies”, “apis cerana”, and “africanized honey bee”) were likely reflecting true connections. Overall, our findings indicated that core research related to apicultural science remained unchanged in both time periods. Words like “geolocation” (the term assigned to refer to geographical locations and environmental factors) acted as a connector between different research foci in both time periods, indicating that the interaction between the honey bee and the environment was related to many other concepts regardless of time period [51,52].

Although the year 2006 was not the most significant split in the cluster analysis, the network graphs (Figure 3 and Figure 4) indicated connections between CCD and the genome release. For example, our post-2006 keyword network showed the co-occurrence of words related to genomic tools (RT-PCR and genome) and health (virus and immunity), which suggested that genomic tools were increasingly used to study honey bee health-related topics [14,53,54]. For example, genomic tools such as gene expression analysis facilitated understanding disease susceptibility, social immunity mechanisms, and response to environmental stressors [55,56]. Similarly, sequencing and RT-PCR have helped to sequence and detect pests and pathogens that impact honey bee health [14,57,58].

In addition to author-assigned keywords, the title and abstract provided more information, leading potentially to better insights and hidden information contained in the full articles. However, the increasing amount of information was more challenging to analyze manually. We used computer-based topic modeling to form the topics, which could be visualized conveniently by the LDAvis interactive tool. The results showed that many distinct, recognizable topics were extracted by the computer even though many keywords were shared among different topics.

The network plot generated from topic modeling showed subtle differences between the pre- and post-2006 time periods. The sharing of words in topics was more prominent in the earlier time period than in the later one, apparent as the connection density, which supported the findings of our keywords’ network analysis. The topic modeling networks also suggested that the research had become more comprehensive and specialized after 2006. There may be multiple reasons for this observation. Increasing specialization has promoted the research topics related to colony losses in the USA and other countries [44,45], which may also have compartmentalized the science. Another reason could be the significant improvement and developments in genomic and molecular tools in science [14,54], opening new subfields. Our stringent selection of keywords in these network analyses (only 20 topics were chosen and further limited the words to the top five words in each topic) may also have obscured some connections between lower-tier keywords that were shared between these topics.

The comparison of networks obtained from author-assigned keywords to topic model keywords could provide information of the authors’ adequacy in describing their overall research in a limited number of keywords. The considerable overlap between nodes was remarkable, but it was clear that topic modeling extracted additional information specifically regarding the connections between topics. Another extension of topic modeling for further improvement could be performed on the entire text rather than on select elements of the articles. Based on the current data, the node overlap increased from the pre-2006 to the post-2006 period, suggesting that authors may have become narrower in their research topics, as well as better at describing their research with relevant keywords over time.

## 5. Conclusions

In conclusion, our findings suggested that the author-assigned keywords were a decent representation of the research articles. However, computational methods could provide additional information, such as connections between some topics that might be missed by manual reading. This study showed that the year 1991–1992 had a major impact on shifting the honey bee research paradigm compared to the general perception of 2006 being the most influential year. However, CCD and honey bee genome release did fuel the bee research mostly comprised of immunity and health topics. The development of new tools and concepts and the need for practical applications induced clear transitions in honey bee research over time. These transitions were slower than anticipated and indicated that the majority of new research foci formed slowly, perhaps reflecting a healthy compromise between continuity and innovation.

## Data Availability

The data and code used to generate the results reported in this manuscript are publicly accessible via a Creative Commons Attribution 4.0 International license at https://doi.org/10.5281/zenodo.3379018.

## Supplementary Materials

The following are available online at https://www.mdpi.com/2306-7381/7/2/61/s1: Figure S1: Temporal trends of selected keywords are shown over the period of 1957–2017, Figure S2: Heat map for the usage of top aggregated keywords over year cluster from the 1957–2005 and 2006–2017 time periods, Figure S3: Snapshot of the interactive LDAvis presentation of the topics (HTML file to explore and visualize topics and relevant keywords generated by topic modeling with LDAvis interactive tools for the time period 1957–2005 can be found at https://doi.org/10.5281/zenodo.3379018: “TopicModelingCorpusPre200620LDA”, as well as for time period 2006–2017: “TopicModelingCorpusPost200620LDA”.), Table S1: Confidence Intervals for transitivity values obtained through bootstrapping over 1000 iteration, Table S2: Example topics and associated salient words corresponding to specific research foci from the pre-2006 time period, Table S3: Example topics and associated salient words corresponding to specific research foci from the post-2006 time period.

## Abbreviations

CCD	Colony Collapse Disorder
CI	Confidence Interval
LDA	Latent Dirichlet Allocation
PCR	Polymerase Chain Reaction
RT-PCR	Reverse Transcription Polymerase Chain Reaction
