# Supplementary material for: Using Manual and Computer-Based Text-Mining to Uncover Research Trends for Apis mellifera
Source: Vet Sci. 2020 May 6;7(2):61. doi: 10.3390/vetsci7020061 (PMC7356030; doi:10.3390/vetsci7020061)
Supplement: Supplementary file 1 [file vetsci-07-00061-s001.pdf]

Supplementary Figures

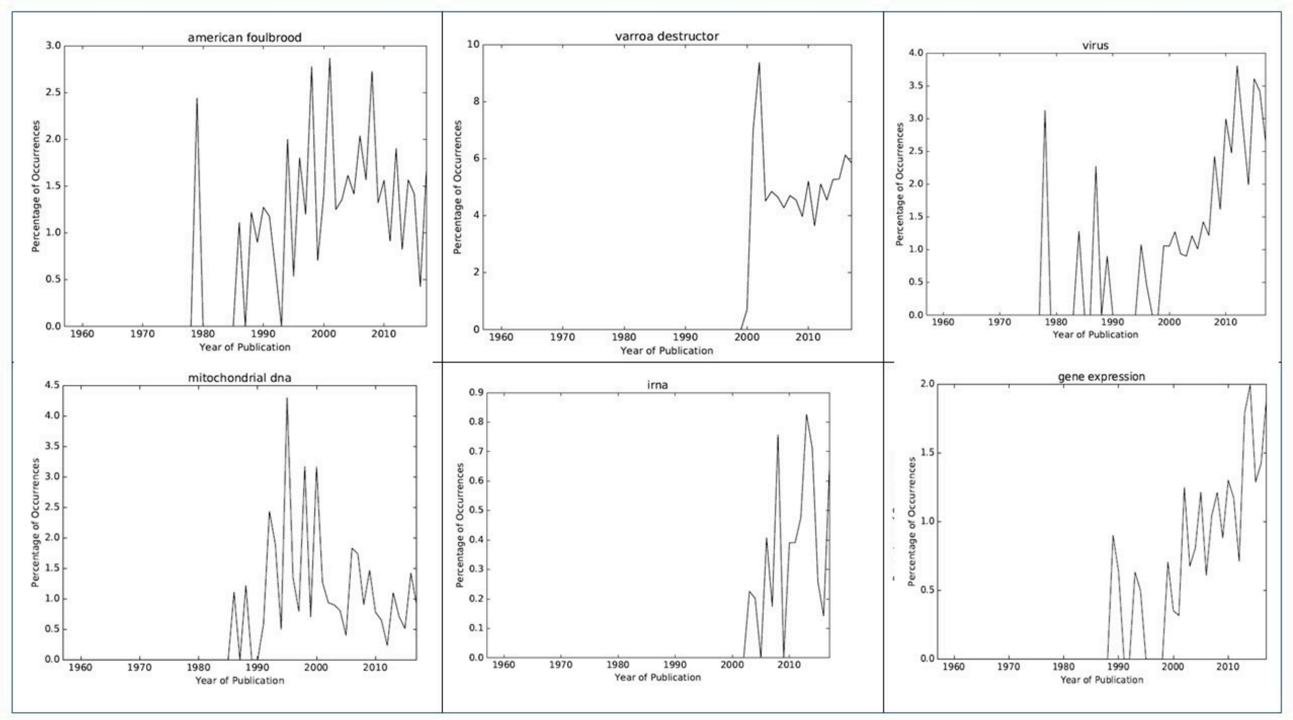

Figure S1. Temporal trends of selected keywords are shown over the period of 1957-2017. The chosen examples are from two major honey bee sub fields of Health (american foulbrood, virus, and varroa destructor) and Genomics (gene expression, irna, and mitochondrial dna) and represent three fundamental patterns: an increase followed by a decrease (left side), an abrupt increase without prior occurrences (center) and a gradual increase (right side).

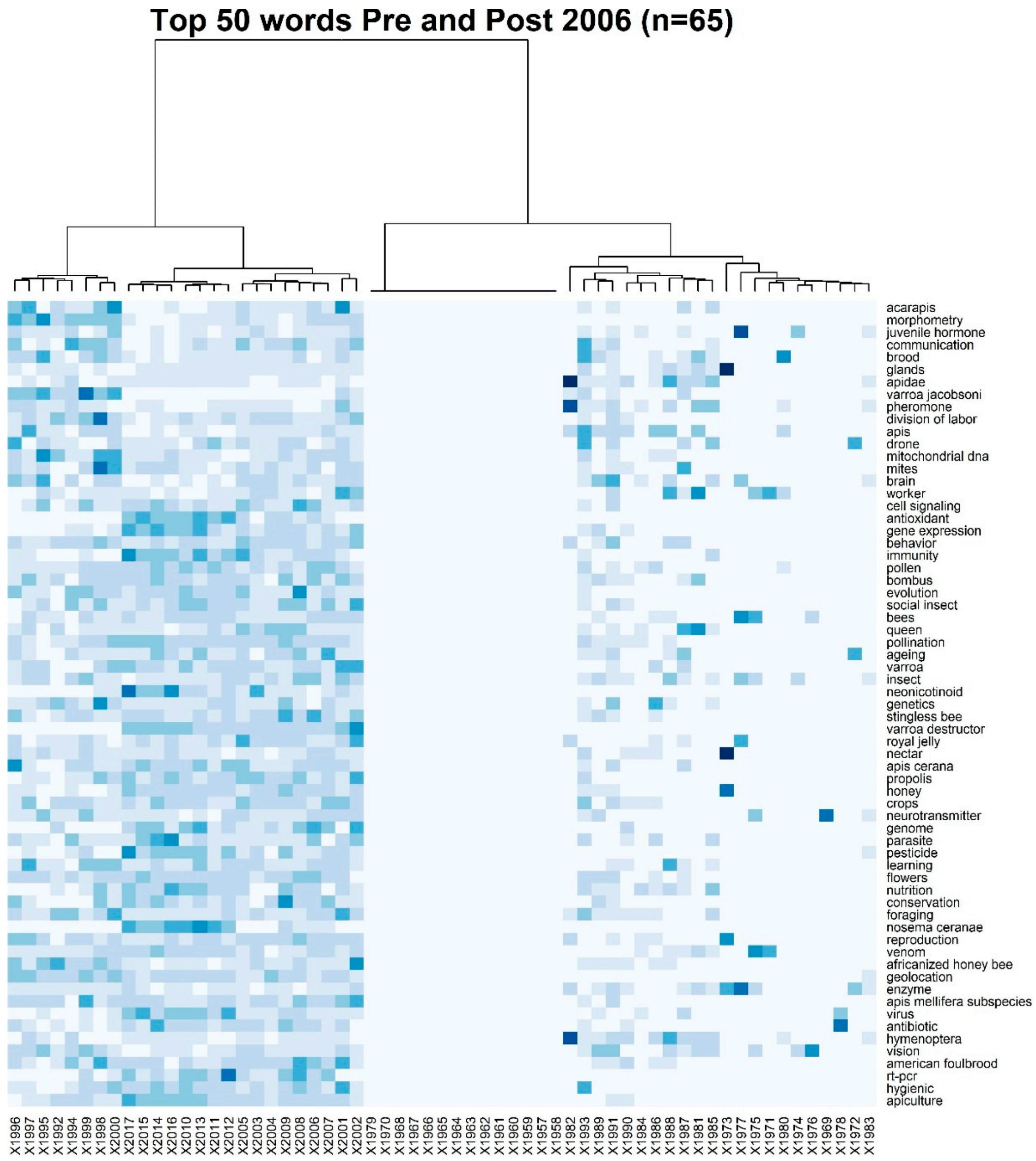

Figure S2. Heat map for the usage of top aggregated keywords over year cluster from 1957-2005 and 2006-2017 time periods. The dark color denotes higher usage while lighter color denotes less usage of the words.

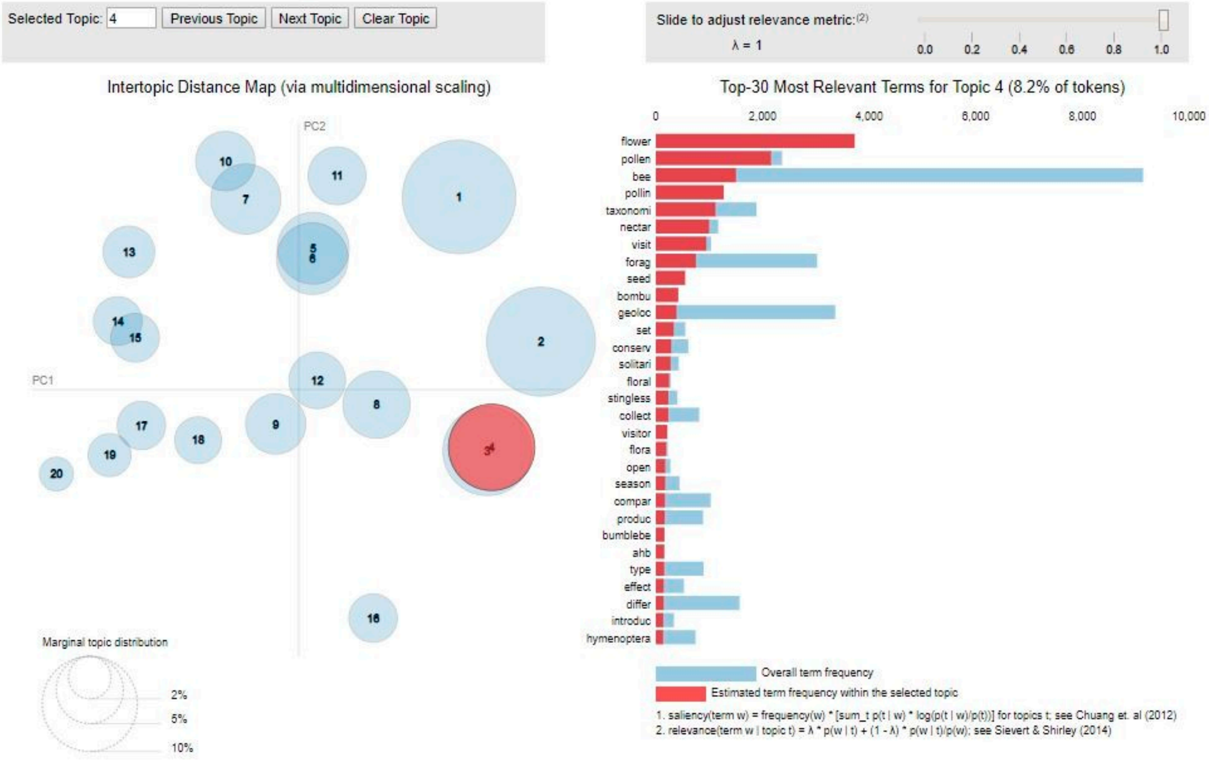

Figure S3. Interactive LDAvis presentation of the topics. The left side of the Fig shows the distribution of top 20 topics on an intertopic distance map. The right side of the Fig lists down the top 30 most relevant terms of that particular topic. This Fig shows an example of selecting a topic (in this case, topic 4) on the left-hand side of this tool, highlighting automatically the most relevant terms for interpreting the selected topic on the right-hand side. The highlighted topic in the Fig appears to address pollination related terms.

Supplementary Tables

Table S1: Confidence Intervals for transitivity values obtained through bootstrapping over 1000 iteration

Network plot	Transitivity	95% CI (for 1000 iteration)
Figure 3	0.5281208	(0.5281151, 0.5281227)
Figure 4	0.437247	(0.4372381, 0.4372498)
Figure 5	0.795756	(0.7953269, 0.7960882)
Figure 6	0.7092199	(0.7089974, 0.7097561)

Table S2: Example topics and associated salient words corresponding to specific research foci from the pre-2006 time period.

Topic Number	Selected relevant words	Research focus
**1**	Bee, foraging, dance, test, model, pattern, fruit, communication, language, discrimination	Behavior
**3**	Geolocation, mite, varroa, african, brood, bee, subspecies, destructor, apiculture, jacobsoni	Varroa
**4**	Flower. Pollen, bee, pollination, taxonomy, nectar, visit, forage, seed, bombus	Pollination
**5**	Gene, sequence, dna, genetics, genotype, mitochondria, marker, genome, pcr, taxonomi	Genomics
**6**	Queen, pheromone, egg, reproduction, larvae, gland, ovary, cast, produce, cell	Reproduction

Table S3: Example topics and associated salient words corresponding to specific research foci from the post-2006 time period.

Topic Number	Selected relevant words	Research focus
**7**	Gene, sequence, genome, dna, taxonomy, rna, epigenetic, pcr, identification, transcriptome	Genomics
**12**	Ceranae, nosema, infection, pathogen, parasite, bee, spore, geolocation, colony, prevalence	Nosema infection
**14**	Subspecies, geolocation, genetics, african, marker, mitochondria, morphology, dna, hybrid, breed	Population
**17**	American, foulbrood, antibiotic, larvae, oil, isolation, strain, vitro, bacteria, extract	Bacterial infection
**18**	Virus, DWV, odor, morphology, infection, viral, olfactory, bee, pcr, transmission	Virus infection
